# The relationship between stigma and psychological distress among people with diabetes: a meta-analysis

**DOI:** 10.1186/s40359-023-01292-2

**Published:** 2023-08-24

**Authors:** Xiajun Guo, Sijia Wu, Haishan Tang, Yuanyuan Li, Wanglin Dong, Guangli Lu, Shuang Liang, Chaoran Chen

**Affiliations:** 1 Institute of Nursing and Health, School of Nursing and Health, Henan University, Kaifeng, 475004 Henan China; 2 Institute of Business Administration, School of Business, Henan University, Jinming Avenue, Kaifeng, 475004 Henan China

**Keywords:** Diabetes mellitus, Stigma, Psychological distress, Meta-analysis, Review

## Abstract

**Background & aims:**

Diabetes may perceive or experience varying degrees of stigma and psychological distress. The association between diabetes-related stigma and psychological distress has been examined in many studies, but no research has used a quantitative synthesis method to investigate the severity of this association and the moderators of the relationship. Thus, we conducted a meta-analysis to quantitatively integrate previous findings to identify the magnitude of the association between stigma and psychological distress among people with diabetes.

**Review methods:**

Following the Preferred Reporting Items for Systematic Reviews and Meta-Analyses (PRISMA) 2020 guidelines, we systematically searched four English academic databases (PubMed, Embase, Web of Science, and PsycINFO) and three Chinese databases (China National Knowledge Infrastructure [CNKI], WANFANG Data, China Science and Technology Journal Database [VIP]). The databases were searched from the inception of each database to the end of March 2023. The pooled correlation coefficient of the association between stigma and psychological distress among people with diabetes was calculated by a random effects model using Stata software (version 17.0), and several moderators that impacted this relationship were identified.

**Results:**

Eligible studies (N = 19) with a total of 12,777 participants were analysed. The pooled correlation was high between diabetes-related stigma and psychological distress (r = 0.50, 95% CI: [0.43–0.57]). Moreover, the association was moderated by the diabetes stigma measurement tools and diabetes distress measurement tools used. However, the relationship was not moderated by type of diabetes, age, gender, geographical location, or type of stigma.

**Conclusions:**

The results of the meta-analysis showed that stigma is strongly related to psychological distress among people with diabetes. Longitudinal or experimental research should be expanded in the future to further identify the causal pathways in the relationship between diabetes stigma and diabetes distress.

**Supplementary Information:**

The online version contains supplementary material available at 10.1186/s40359-023-01292-2.

## Introduction

Diabetes mellitus is a lifelong metabolic disease characterized by chronic hyperglycaemia due to a combination of abnormal insulin secretion and/or defective insulin utilization and multiple factors [1]. The International Diabetes Federation (IDF) Diabetes Atlas (10th edition) showed that 537 million adults are currently living with diabetes worldwide, and if this trend continues, this number is expected to increase to 783 million people by 2045 [2]. This disease requires long-term treatment and self-management to control blood glucose, slow disease progression, and reduce the risk of complications [3]. However, diabetes may suffer from varying degrees of social discrimination due to stereotypes of diabetes (e.g., obesity, poor dietary habits, and sedentary lifestyles) by the public and the patients themselves [4]. In addition, when people with diabetes need to inject insulin, monitor their blood glucose, or take medications in public places, they often receive strange looks from others, which can lead to negative psychological experiences such as shame and stigma [5].

The sociologist Goffman [6] first cited the concept of stigma in social psychology, defining it as “features that greatly tarnish someone’s reputation”. The concept of stigma has been gradually applied to the medical field, from mental diseases [7] and infectious diseases [8] to various chronic diseases [9, 10]. Diabetes stigma refers to the experience of negative feelings of self-blame, shame, and exclusion by individuals with diabetes who are labelled to distinguish them from others or even to devalue them because they have the disease [10]. A recent large multinational survey showed that one in five (19.2%) patients with diabetes reported experiencing discrimination [11]. To avoid social discrimination, most diabetes will take certain measures to conceal their disease, such as delaying or missing insulin injections [12], not monitoring their blood glucose in places other than home [13], and refusing to participate in social activities such as dining together, which seriously affect their treatment compliance and quality of life [14]. Stigma has become one of the barriers to self-management in patients with diabetes [15]. Currently, the International Diabetes Federation has declared that diabetes-related stigma requires exigent attention, with a necessity to “champion a world free from discrimination and stigma for people with diabetes” [16].

Due to stigmatization, diabetes might have an increased risk of mental health problems (e.g., psychological distress and depression) [17, 18]. At present, some researchers have investigated the relationship between stigma and psychological distress among patients with diabetes [19–21]. Diabetes-related psychological distress is a negative emotional condition experienced by patients due to concerns about diabetes self-management, social support, and disease treatment effects [22]. Polonsky et al. [23] noted that diabetes-specific psychological distress mainly includes distress related to changes in life patterns, an increased emotional burden, medical treatment, and interpersonal communication. According to a systematic review, the overall prevalence of psychological distress was 36% in patients with diabetes [24]. Diabetes-related distress is not only a psychological burden on patients but can also negatively impact their diabetes-related health outcomes [25] and quality of life as well as blood glucose control [26, 27]. In a qualitative study of diabetes patients’ perspectives on distress, a proportion of patients expressed that diabetes stigma discriminated them from others and that this awareness was an important cause of aggravated diabetes distress [28].

Overall, diabetes-related stigma has an important effect on the outcomes of biopsychosocial health for diabetes patients [14, 29, 30]. Recently, a systematic review also reported that diabetes-related stigma is negatively related to clinical, psychological, and behavioural outcomes in patients with type 2 diabetes [31]. However, diabetes-related psychological distress is not only a burden on patients’ spirituality but also hinders patients’ self-management of the disease, thus affecting glycaemic control [32]. Therefore, determining the correlation between diabetes stigma and diabetes-related distress will further inspire researchers to focus on the psychosocial aspects of diabetes and enable better psychological care for patients. Many previous empirical studies have reported the relationship between diabetes stigma and psychological distress [33–35], but no research has used a quantitative synthesis method, such as meta-analysis, to investigate the severity of this association and moderators of the relationship. Additionally, the magnitude of the correlation has been a controversial topic [19, 36]. Thus, we utilized a meta-analysis to integrate the results of previous empirical studies on the relationship between diabetes stigma and psychological distress to identify the magnitude and potential mediators of the relationship between the two variables, thereby laying the groundwork for further improvements in the mental health of people with diabetes.

In this work, we examined whether the association between stigma and psychological distress among people with diabetes was moderated by some factors, such as (a) the type of diabetes of the sample, (b) the diabetes-related stigma and diabetes-specific psychological distress measurement tools used, (c) the type of stigma, and (d) the demographic characteristics of the sample (age, gender, geographical location). First, diabetes mellitus is not a single disease; it encompasses several broad types (e.g., type 1 diabetes [T1D], type 2 diabetes [T2D], gestational diabetes, and other special types of diabetes mellitus) that differ in their manifestations, etiology, and management, which is reflected in the different perceptions of diabetes-related stigma [4, 5, 37]. For example, although both patients with T1D and T2D report stigma, patients with T1D probably report higher levels of negative judgments and emotional distress than patients with T2D due to the need for lifelong treatment with insulin injections [38]. Thus, we hypothesized that the correlation between diabetes-related stigma and psychological distress may be moderated by the type of diabetes (Hypothesis 1). Second, considering the different number of items and dimensions of each scale, we hypothesized that the instruments used to measure diabetes stigma and psychological distress might moderate the relationship between stigma and psychological distress in people with diabetes (Hypotheses 2 and 3, respectively). Third, stigma is classified into perceived stigma (an individual’s perception of stigmatization and awareness of the stereotype and discrimination against their characteristics), experienced stigma (the experience of stigmatization, prejudice, and discrimination from others), and self-stigma (internalized stigma, refers to individuals who have accepted the bias and discrimination against their characteristics) at the individual level [39, 40]. One previous study showed that once stigma is internalized, it may be harder to avoid the psychological distress related to stigma [41]. Thus, we hypothesized that the relationship between diabetes-related stigma and psychological distress could be moderated by the type of stigma (Hypothesis 4). Additionally, Graue et al. [42] found that the diabetes-related distress score was negatively correlated with age, with participants’ emotional burdens and life regularity-related distress decreasing with age. This may be because young patients are usually the main source of economic income for their families, and the disease can limit patients’ social intercourse and work to some extent, so younger patients are prone to more severe psychological distress. Given this, we hypothesized that the relationship between diabetes stigma and psychological distress could be moderated by age (Hypothesis 5). Furthermore, female patients with diabetes are more likely to develop diabetes-related psychological distress than male patients [42]. A recent meta-analysis confirmed that gender is a moderator of the association between weight stigma and psychological distress [43]. Therefore, we hypothesized that gender might be a factor moderating the relationship between stigma and psychological distress among people with diabetes (Hypothesis 6).

In summary, the purposes of this study were to synthesize the findings of previous studies that focused on the relationship between stigma and psychological distress among people with diabetes and to examine some moderators that may impact this relationship, which contributes to identifying the source of interstudy heterogeneity and the magnitude of the relationship between the two variables.

## Methods

### Study design

The present study was conducted following the PRISMA guidelines [44]. In addition, the protocol of the study was registered in PROSPERO—an international prospective registry of systematic reviews (reference code: CRD42023413726).

### Search strategy

We searched four English databases (PubMed, Web of Science, Embase, and PsycINFO) and three Chinese databases (CNKI, VIP, and WANFANG Database) from inception to March 2023. We combined search terms using relevant MeSH terms and the free terms of diabetes mellitus, stigma, and psychological distress. After that, the appropriate Boolean operators (AND, OR, NOT) were selected to combine the search terms. The detailed search strategies for all databases are available in **Supplementary File 1**. Moreover, the reference lists of the included articles and related systematic reviews were also examined by hand to prevent the omission of potential studies.

### Study selection criteria

The retrieved studies were scrutinized independently by two reviewers following the inclusion/exclusion criteria listed below. Any disputes were resolved by a third senior researcher. The inclusion criteria were (1) studies including participants who were diagnosed with diabetes; (2) studies using valid quantitative measures to evaluate stigma and psychological distress among people with diabetes; (3) studies containing quantitative data that identified the statistical association between diabetes-related stigma and psychological distress; and (4) primary studies if duplicate publications were reported for the same samples. The exclusion criteria were (1) studies not published in English or Chinese and (2) conference reports, editorials, and letters.

### Data extraction

Two authors extracted data independently using a predefined form. The following detailed information was extracted from the included studies: the surname of the first author, year of publication, sample size, country, type of diabetes, proportion of female participants (%), age (mean ± SD), instruments used to measure diabetes stigma levels, instruments used to evaluate diabetes-related psychological distress levels, Pearson’s correlation coefficients between diabetes stigma and psychological distress and type of stigma. Any differences in opinion were initially discussed by the two authors, and further additional differences were settled by a third researcher. If studies reported Spearman correlation coefficient *r* or *F*, *t*, χ^2^ and β values rather than Pearson’s correlation coefficients *r* values, the authors converted them to Pearson’s *r* values based on the following formula: *r* = $2{sin}\left({r}_{s}\frac{\pi }{6}\right)$ [45], *r* = $\sqrt{\frac{{t}^{2}}{{t}^{2}+df}}$, *r* = $\sqrt{\frac{F}{F+d{f}_{e}}}$, *r* = $\sqrt{\frac{{\chi }^{2}}{{\chi }^{2}+N}}$, *r* = β × 0.98 + 0.05 (β ≥ 0); *r* = β × 0.98 − 0.05 (β < 0) [-0.5 < β < 0.5] [46].

### Quality appraisal

In the present study, a cross-sectional assessment provided by the Agency for Healthcare Research and Quality (AHRQ) was used to evaluate the quality of the included studies [47]. The assessment included a total of 11 specific items. Two researchers independently assessed the quality of the included studies, and a third senior researcher was responsible for resolving any disagreements or disputes. Articles were given a score of “1” if they met the requirements of the item; a score of “0” was given for responses of “No” or “Unclear”. The following criteria were used to evaluate the quality of the articles: low quality (0–3 points), moderate quality (4–7 points), and high quality (8–11 points).

### 2.6 Statistical analysis

Stata software (version 17.0) was used to analyse all of the data. The target effect size for the meta-analysis was identified by the quantitative data about the association between diabetes stigma and psychological distress, reported as Pearson correlation coefficients. Correlation coefficients might influence the variance, so the correlation coefficient for each included study was transformed to Fisher’s z values, and all analyses were conducted with Fisher’s z values as the effect size [48]. The following formula was used to convert correlation *r* values to Fisher’s z values: z = 0.5 × ln [(1 + r)/(1 – r)]. In addition, the variance of z was calculated by the equation Vz = 1/(n – 3), and the standard error of z was calculated by the equation SEz = $\sqrt{1/(n-3)}$, where n is the sample size.

Given that the included studies could be performed in a variety of settings, we chose the random effect model and Der-Simonian and Laird’s method to perform this meta-analysis since it accounts for study heterogeneity [49]. To determine the heterogeneity, we used both Cochran’s Q test and the I-squared statistic [50]. *I*^2^ values of less than 25%, 50%, and more than 75% indicated low, moderate, and high heterogeneity, respectively (explaining with caution) [51]. A significant degree of heterogeneity (*I*^2^ > 75%, p < 0.05) indicated that there were potential moderating effects among the included papers. Subgroup analysis and meta-regression were used to conduct the moderator analysis. Potential moderators included the type of diabetes, geographical location of the research (by continent), scales used for diabetes-related stigma, measures used for diabetes-specific psychological distress, the type of stigma, age (mean), and gender (the proportion of female participants).

In addition, funnel plots and Egger’s linear regression test were used to assess publication bias [52]. The trim-and-fill method was used to correct the results in the case of possible publication bias [53]. Finally, the jackknife method (also known as the ‘leave-one-out method’) was used for sensitivity analysis.

## Results

### Search results

**Fig. 1:**
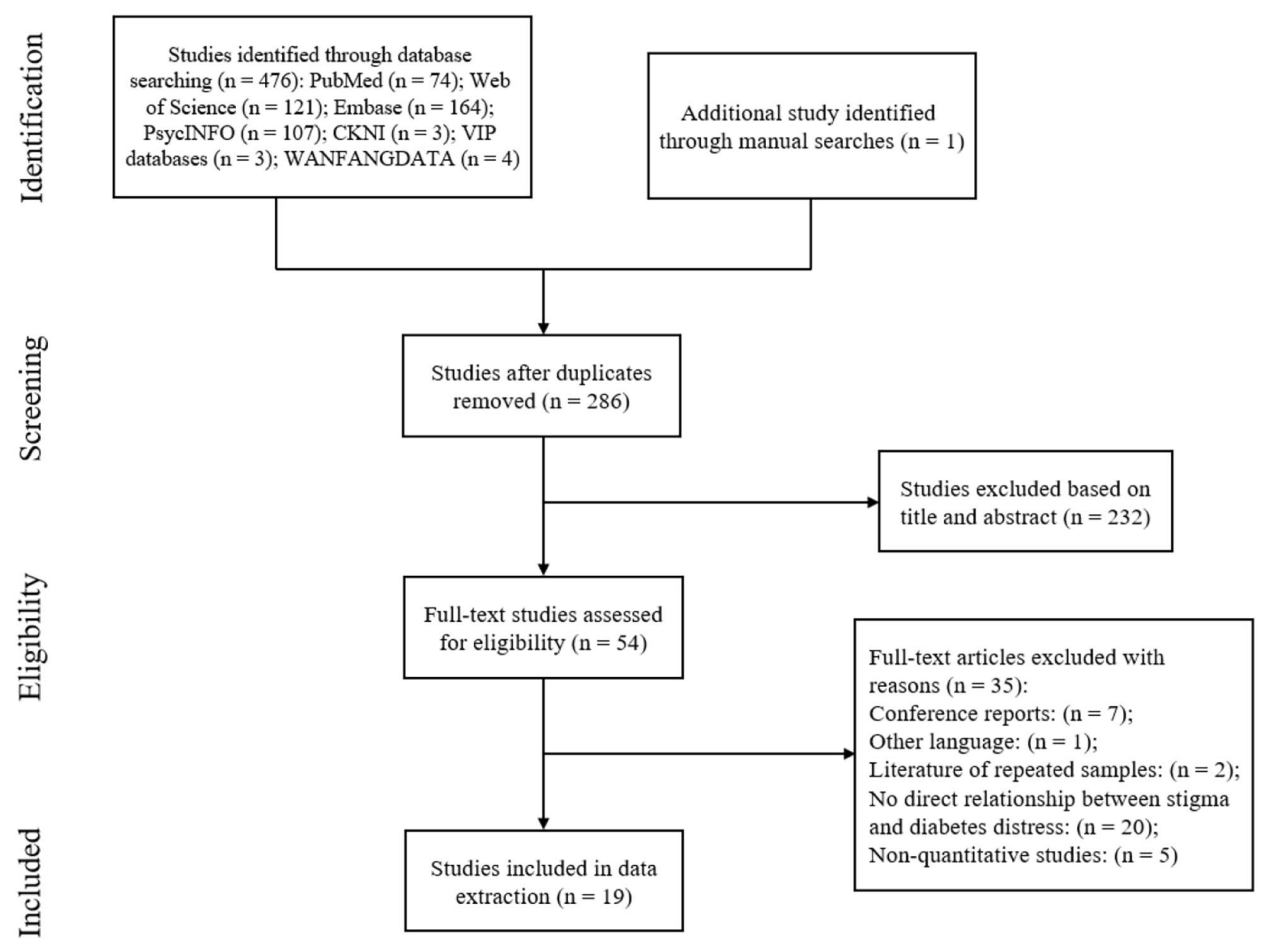
Flow chart of study selection according to the PRISMA guidelines

According to our search strategy, a total of 477 studies were preliminarily identified in the databases, and one study was found through manual searches. After removing duplicates, 286 articles remained, of which 232 were excluded after reviewing the titles and abstracts because they were not in accordance with the topic. Then, the reviewers evaluated the complete texts of 54 studies considered potentially relevant. The full texts of 54 studies deemed to be potentially relevant were evaluated by the reviewers. Of these, 35 articles were excluded because they did not report the direct relationship between stigma and diabetes-specific psychological distress (n = 20) or because they were conference reports (n = 7), were nonquantitative studies (n = 5), had repeated samples (n = 2), or were published in other languages (n = 1). Ultimately, the meta-analysis included 19 articles that met all inclusion criteria. Figure 1 shows the literature screening procedure following the PRISMA guidelines.

### Characteristics of the included studies and quality assessment

All the included studies were published from 2015 to 2022 and had a cross-sectional design (Table 1). A total of 12,777 patients with T1D or T2D were included in the 19 studies, with the sample size ranging from 105 to 3,347 in each study. Regarding the included studies’ geographic locations, 3 studies were from Australia, 5 studies were from the USA, 5 studies were from China, and 6 studies were from Denmark, United Arab Emirates, Colombia, Korea, Switzerland, and Greece. The measure used for evaluating stigma mainly included the Type 1 Diabetes Stigma Assessment Scale (DSAS-1), the Type 2 Diabetes Stigma Assessment Scale (DSAS-2), the Self-Stigma Scale (SSS), the Perceived Stigma Measurement (PSM) tool, the Everyday Discrimination Scale (EDS), etc. In addition, the most frequently used measures for evaluating diabetes-specific distress mainly included the Problem Areas in Diabetes (PAID) scale, the 5-item Problem Areas in Diabetes (PAID-5) scale, the Diabetes Distress Scale (DDS), etc. All scales used in the included studies showed good reliability and validity. Because of the different implications of perceived stigma, experienced stigma, and self-stigma, we also categorized the eligible studies according to the type of stigma. Of the 19 studies, 5 evaluated diabetes self-stigma, and 14 evaluated diabetes-related perceived and experienced stigmas. Finally, according to the study appraisal results, the included studies were of moderate-to-high quality. **Supplementary File 1** contains the details of the quality evaluation of the included studies. In addition, the characteristics of the included studies are shown in Table 1.

**Table 1:** Characteristics of the 19 studies included in the meta-analysis

Author, year	Country	n	Type of diabetes	Female (%)	Age: Mean ± SD	Stigma Scale	Distress Scale	r	Type of stigma
Hansen et al., 2017 [54]	Denmark	1594	1	49.81	49 ± 13.6	DSAS-1	PAID-5	0.51	PES
Alzubaidi et al., 2022 [55]	Arab	327	2	55.96	N/A	DSAS-2	PAID-5	0.38	PES
Browne et al., 2017 [56]	Australia	900	1	59.11	43.87 ± 15.32	DSAS-1	PAID	0.65	PES
Browne et al., 2016 [34]	Australia	1064	2	43.00	61.2 ± 9.4	DSAS-2	PAID	0.69	PES
Li et al., 2022 [57]	China	258	2	51.16	61.98 ± 12.69	SSS	DDS	0.34	SS
Pedrero et al., 2021 [19]	Colombia	501	2	63.30	60 ± 12	DSAS-2	PAID-5	0.20	PES
Potter et al., 2015 [58]	USA	185	2	65.00	55.42 ± 10.1	EDS	PAID	0.38	PES
Hyesun et al., 2022 [35]	Korea	187	All	21.90	62.53 ± 5.2	SSM	PAID	0.71	SS
Puhl et al., 2020 [59]	USA	1227	2	51.40	52.04 ± 14.96	DSAS-2	PAID	0.39	SS
Holmes-Truscott et al., 2020 [38]	Australia	642	2	45.48	61 ± 9.7	DSAS-2	PAID	0.68	PES
Gredig et al., 2016 [17]	Switzerland	3347	All	45.22	64.4 ± N/A	PSM	PAID	0.41	PES
Benioudakis et al., 2022 [60]	Greek	105	1	70.48	34.3 ± 11.1	DSAS-1	DDS	0.44	PES
Polonsky et al., 2021 [36]	USA	599	2	66.78	63 ± 10.5	DDSS	DDS	0.67	SS
Costabile et al., 2020 [61]	USA	399	All	71.68	47.22 ± 14.67	PSM-3	DDS-3	0.29	PES
Wang et al., 2021 [20]	China	193	All	N/A	52.79 ± 8.55	SSS	PAID-S	0.41	SS
Joiner et al., 2022 [62]	USA	517	2	72.34	53.9 ± 10.1	DSAS-2	DDS	0.59	PES
Kong et al., 2022 [21]	China	209	2	N/A	N/A	DSAS-2	DDS	0.38	PES
Li et al., 2021 [33]	China	244	2	41.80	N/A	DSAS-2	DDS	0.69	PES
Xie et al., 2020 [63]	China	279	2	54.12	64.02 ± 9.78	DSAS-2	DDS	0.44	PES

N/A, Not reported; DSAS-1, Type 1 Diabetes Stigma Assessment Scale; DSAS-2, Type 2 Diabetes Stigma Assessment Scale; SSS, Self-Stigma Scale; EDS, Everyday Discrimination Scale; SSM, Self-Stigma Measurement tool; PSM, Perceived Stigma Measurement tool; DDSS, Diabetes-related Emotional Distress Source Scales-shame/stigma; PAID, Problem Areas in Diabetes scale; PAID-5, 5-item Problem Areas in Diabetes; DDS, Diabetes Distress Scale; DDS-3,3-item Diabetes Distress Scale; PAID-S, Short-form Problem Areas in Diabetes scale; PES, Perceived and Experienced Stigma; SS, Self-Stigma.

### Homogeneity tests and pooled effect size

**Fig. 2:**
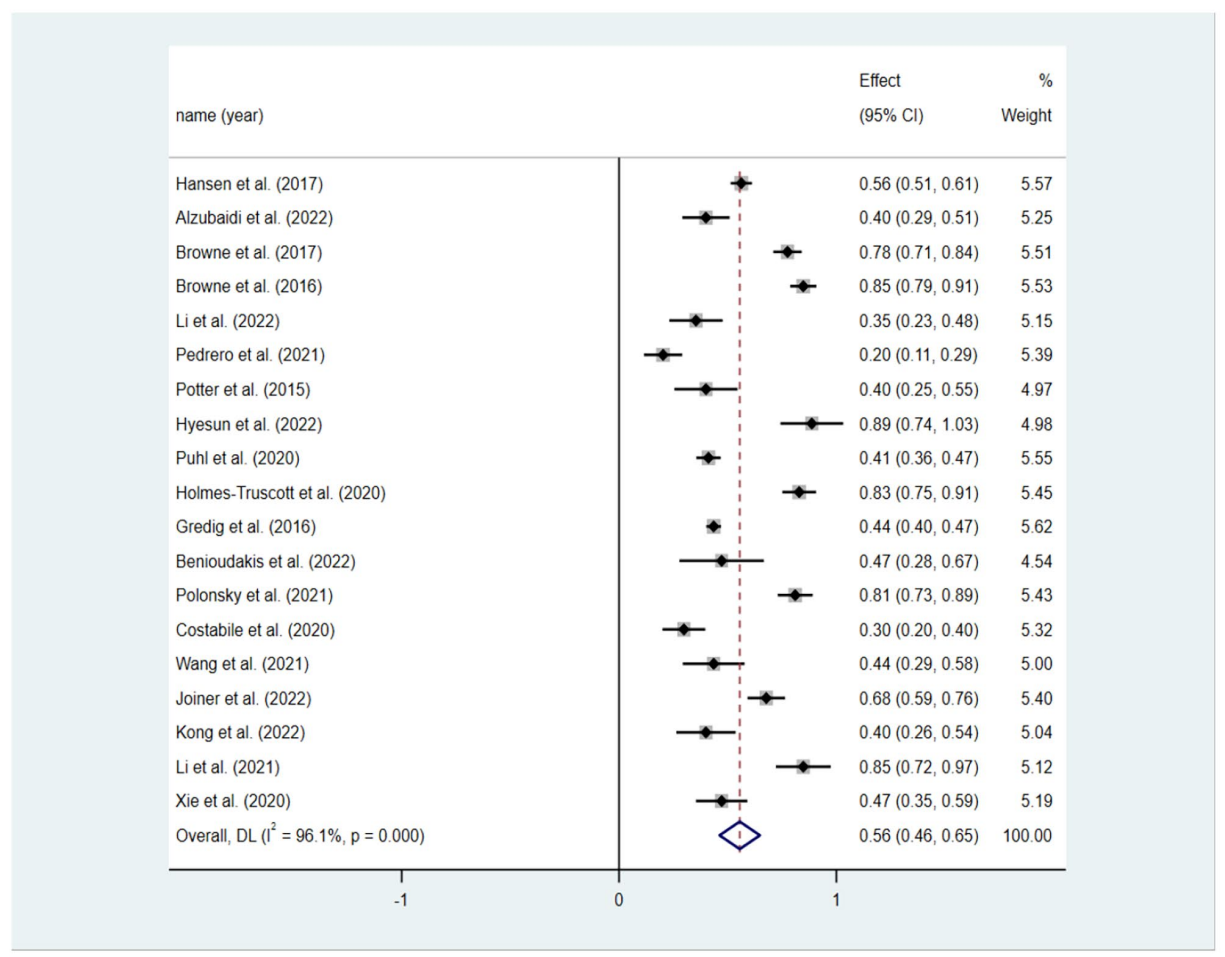
Forest plots for the relationship between diabetes-related stigma and distress

The homogeneity test showed high heterogeneity among all eligible studies (Q-statistic = 463.64; *P* < 0.001; *I*^2^ = 96.10) (shown in Table 2). This high heterogeneity was expected due to the differences in cultural backgrounds and variations in the measures used to assess diabetes-related stigma and distress. Moreover, mild to high correlations between stigma and diabetes-specific distress was found across all included studies (n = 19). The result of the random effects model showed a pooled correlation coefficient of 0.50 (95% CI: 0.43–0.57) between diabetes stigma and psychological distress. As recommended by Lipsey and Wilson [64], Pearson correlation coefficient *r* values of 0.10, 0.25, and 0.40 were regarded to indicate mild, moderate, and high correlations, respectively. Consequently, there was a high positive correlation between stigma and psychological distress among patients with diabetes. Moreover, as shown in Table 2, the correlation between diabetes stigma and psychological distress was stable, with a Z value of 11.72 (*P* < 0.001). Finally, the forest plots for the relationship are shown in Fig. 2.

**Table 2:** Random model of the correlation between diabetes-related stigma and distress

K	N	Effect size (r)	95% CI for r	Homogeneity test				Test of null (two-tailed)	
				**Q (r)**	***P***	***I*** ^**2**^		**Z-value**	***P***
19	12,777	0.50	[0.43, 0.57]	463.64	<0.001	96.10%		11.72	<0.001

### Moderator analysis

The moderator analysis, using analysis of variance (ANOVA) tests, showed that the instruments used to assess diabetes stigma (Q_BET_ = 6.95, df = 2, p < 0.05) and the instruments used to evaluate diabetes distress (Q_BET_ = 9.68, df = 3, p < 0.05) significantly moderated the correlation between stigma and diabetes-related distress among people with diabetes (Table 3). For the instruments used to evaluate diabetes-related stigma, the pooled effect size for the correlation coefficient between diabetes-related stigma and distress was significantly higher when the DSAS-1 was used (r = 0.62, 95% CI = [0.44, 0.79]), but the pooled effect size was relatively lower when stigma was measured with the DSAS-2 (r = 0.57, 95% CI = [0.40, 0.73]) or SSS (r = 0.55, 95% CI = [0.44, 0.66]). Regarding the tool for measuring DSD, the PAID scale (r = 0.66, 95% CI [0.49, 0.82]) had the highest correlation coefficient between diabetes patients’ stigma and distress in comparison to the DDS (r = 0.58, 95% CI [0.43, 0.73]), PAID-5 scale (r = 0.39, 95% CI [0.16, 0.62]), and other scales (r = 0.36, 95% CI [0.22, 0.49]). However, the moderating effects of the type of diabetes, geographical location, and type of stigma on the relation between diabetes-related stigma and distress were not significant (all p > 0.05).

In addition, as shown in Table 4, a meta-regression confirmed that gender and age were not significant moderators of the correlation of diabetes-related stigma with distress.

**Table 3:** Stigma and diabetes-specific distress: Univariate analysis of variance for moderators

	Q_BET_	K	N	r	95% CI for r	QW	*I* ^2^
**Type of diabetes**	0.28						
type 1 diabetes		3	2599	0.62	[0.44, 0.79]	28.66***	93.00%
type 2 diabetes		12	6052	0.56	[0.42, 0.70]	313.31***	96.50%
**DSD measures**	9.68*						
PAID-5		3	2422	0.39	[0.16, 0.62]	50.81***	96.10%
PAID		7	7552	0.66	[0.49, 0.82]	277.36***	97.80%
DDS		7	2211	0.58	[0.43, 0.73]	71.77***	91.60%
Others		2	592	0.36	[0.22, 0.49]	2.41	58.50%
**Stigma measures**	6.95*						
DSAS-1		3	2599	0.62	[0.44, 0.79]	28.66***	93.00%
DSAS-2		9	5010	0.57	[0.40, 0.73]	263.61***	97.00%
SSS		2	451	0.55	[0.44, 0.66]	0.72	0.00%
**Geographical location**	2.72						
Europe		2	1699	0.56	[0.51, 0.61]	0.79	0.00%
Asia		7	1697	0.54	[0.38, 0.70]	67.28***	91.10%
Oceania		4	5953	0.72	[0.48, 0.96]	216.66***	98.60%
America		6	3428	0.47	[0.28, 0.66]	143.43***	96.50%
**Type of stigma**	0.07						
Perceived and experienced stigma		14	10,313	0.55	[0.44, 0.66]	366.29***	96.50%
Self-Stigma		5	2464	0.58	[0.36, 0.79]	96.87***	95.90%

**Table 4:** Univariate regression analysis of gender and age (random-effect model)

z	K	Coef.	SE	t	P	95% CI
Female participants (%)	17	-0.008	0.004	-1.99	0.065	[-0.016, 0.001]
_cons		1.004	0.224	4.49	0.000	[0.527, 1.480]
Age Mean	16	0.004	0.007	0.60	0.559	[-0.011, 0.019]
_cons		0.326	0.388	0.84	0.415	[-0.507, 1.159]

### Publication bias

**Fig. 3:**
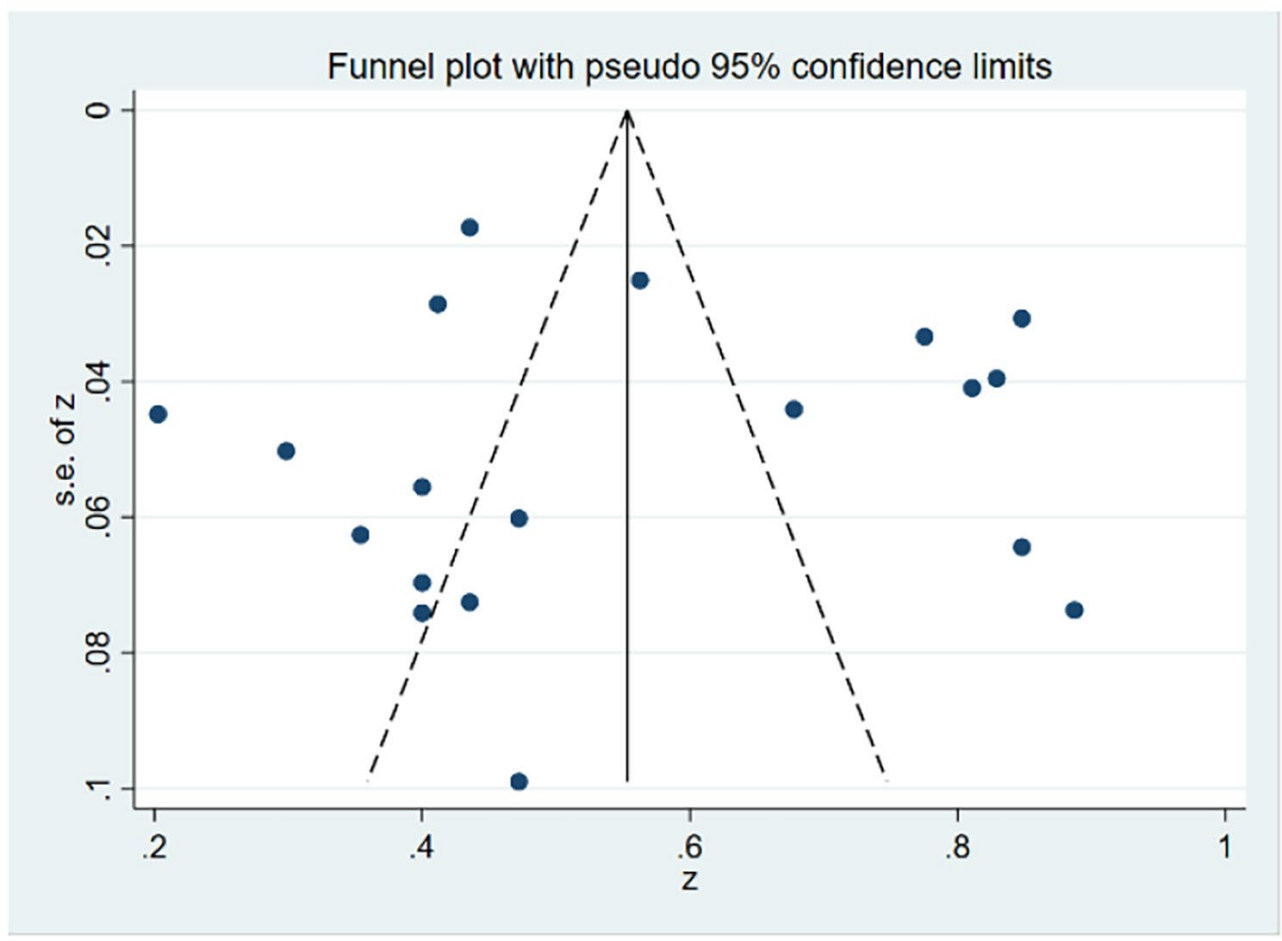
Funnel plots to assess publication bias in the association of diabetes-related stigma with psychological distress

**Fig. 4:**
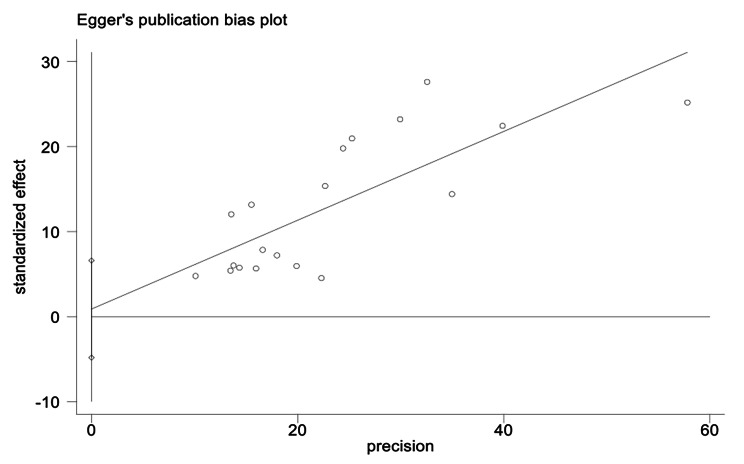
Egger’s test to assess publication bias for the correlation between diabetes-related stigma and psychological distress

**Fig. 5:**
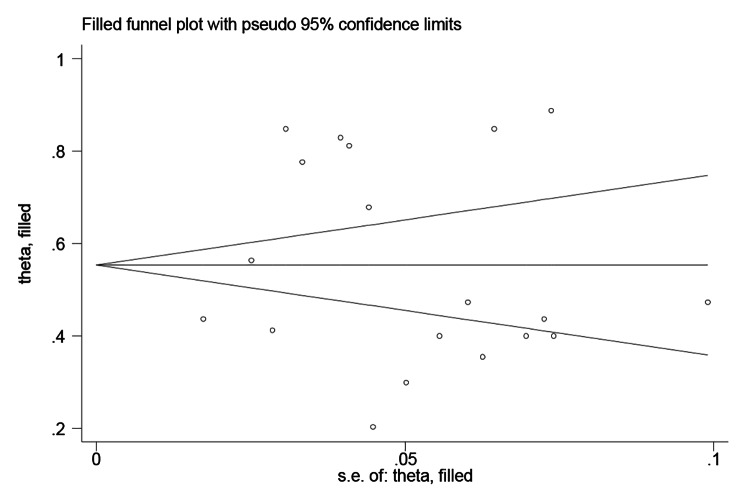
Corrected funnel plots for the correlation between diabetes-related stigma and distress using the trim-and-fill method

First, the funnel plot (Fig. 3) shows that the effect sizes for the association of stigma and diabetes-specific distress among patients with diabetes were approximately evenly distributed on both sides of the estimated overall pooled effect size, implying a small probability of publication bias. Due to the subjectiveness of such a judgment, we used Egger’s test to validate it further. Egger’s regression also showed that there was no significant publication bias (t = 0.32, p = 0.75) (Fig. 4). The trim-and-fill method also identified that no correction was performed, and the data remained unchanged (Fig. 5), indicating that no significant publication bias existed.

### Sensitivity analysis

**Fig. 6:**
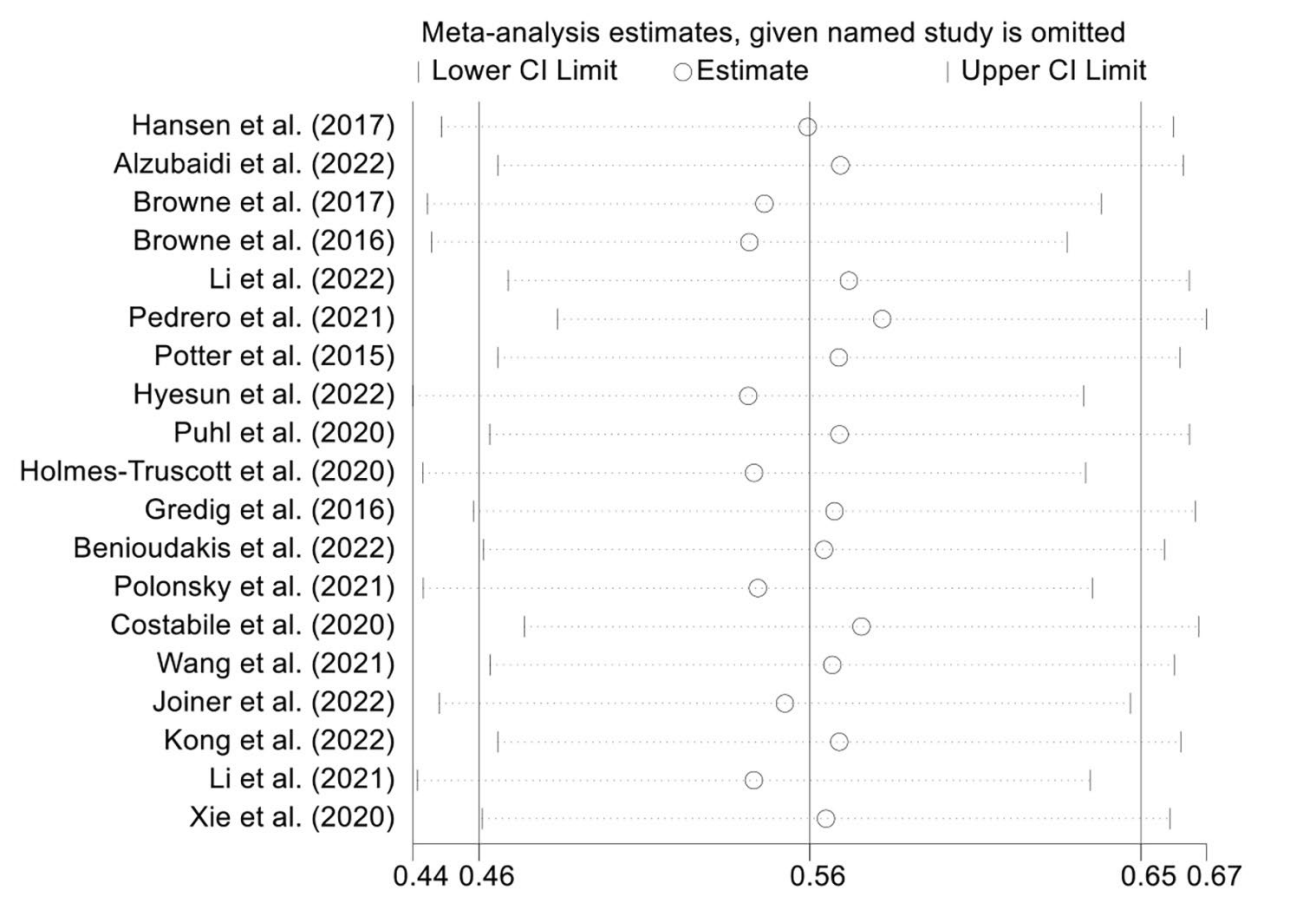
Sensitivity analyses of the 19 studies included in the meta-analysis

After sequentially removing one study in turn and then recalculating the overall correlation coefficients for the remaining studies, only minor changes were found in the results, validating that our findings were stable (Fig. 6).

## Discussion

### Relationship between diabetes stigma and psychological distress

In the past three years, there has been increasing interest in exploring the association between diabetes-related stigma and psychological distress (14 out of the 19 studies were published in 2020–2022) [19–21, 33, 35, 36, 38, 55, 57, 59–63]. After rigorous selection by the PRISMA guidelines, the meta-analysis included 19 studies with a total of 12,777 participants to provide evidence of the association between diabetes-related stigma and psychological distress. This present study is the first to conduct a meta-analysis to quantitatively integrate previous findings to identify the magnitude of the association of stigma with psychological distress among people with diabetes. The results showed a high positive relationship between stigma and psychological distress among people with diabetes (r = 0.50 [95% CI: 0.43–0.57]). This result indicated that higher diabetes-related stigma is significantly associated with increased psychological distress in diabetes. According to the UK’s 2019 diabetes and mental well-being workshop, the impact of stigma on the mental health of diabetes deserves more attention to prevent its negative effects [65]. The stereotypes associated with diabetes are important factors that contribute to the stigma around the disease. These include stereotypes emphasizing inadequate individual responsibility and self-management because diabetes mellitus is deemed a lifestyle disease (e.g., bad dietary habits and sedentary lifestyles) [66]. In addition, diabetes-related stigma could cause a range of negative experiences associated with diabetes treatment or management [67, 68]. These negative experiences are internalized by people with diabetes and lead to the formation of negative emotions and thoughts associated with diabetes distress [28]. Given that social withdrawal is also associated with diabetes stigma [69], it is necessary to pay more attention to diabetes-related interpersonal distress resulting from diabetes stigma. Furthermore, future longitudinal studies are needed to investigate the development of diabetes stigma to psychological distress or, in turn, to explore the trajectory of psychological changes among participants with high levels of diabetes stigma in depth. We hope to encourage health care providers to give attention to diabetes-related stigma and distress and provide appropriate and effective intervention strategies to reduce their negative impact on patients with diabetes.

### Explanations of the moderating effect

The heterogeneity analysis revealed a significant degree of unexplained variation within and between studies. To explore the source of heterogeneity, the moderating effects of the type of diabetes, measurement tools used to evaluate diabetes-related stigma and distress, geographical location, type of stigma, gender, and age on the association between diabetes-related stigma and psychological distress were examined in the present study. The results of the moderating effect analysis showed that the measurement tools used to evaluate diabetes-related stigma and psychological distress were significant moderators. However, inconsistent with our previous hypothesis, these hypothesized moderators (type of diabetes, type of stigma, gender, age, and geographical location) did not influence the relationship.

First, no significant moderating effect of the association by type of diabetes was found. Previous studies also found that regardless of the type of diabetes, the relationship between diabetes-related stigma and distress in diabetes was strong [70, 71]. This might be due to the following reasons: the current meta-analysis included only three studies of patients with type 1 diabetes. Most of the extracted effect sizes were from populations consisting almost exclusively of T2D patients. Thus, the lack of T1D or other types of diabetes patients may be a potential explanation for the absence of a moderating role of the type of diabetes. Second, although patients tend to perceive and experience higher diabetes distress and stigma due to the need for lifelong insulin treatment [29, 72], patients with type 2 diabetes also frequently feel discriminated against and shamed because of their weight and dietary habits [73]. Thus, the difference in the pooled correlation coefficient was not significant. However, due to the lack of several studies included in the subgroup analysis, the results about the moderating effects of the type of diabetes should be interpreted with caution. If research on type 1 diabetes or other types of diabetes is enriched in the future, further meta-analyses should again consider the type of diabetes as a potential moderator.

Second, subgroup analysis found that the measurement tools of diabetes stigma significantly moderated the magnitude of the association between stigma and diabetes distress in patients with diabetes. This positive correlation was significantly higher when stigma was measured with the DSAS-1 (r = 0.62) than with the DSAS-2 (r = 0.57) or SSS (r = 0.55). Possible reasons for the difference may be that the DSAS-1 and DSAS-2 are suitable for different subjects of study (measuring T1D patients and T2D patients, respectively), and each scale has different measuring characteristics (the DSAS-1 and DSAS-2 measure perceived and experienced stigma, and the SSS mainly measures self-stigma) [19, 20, 56]. Additionally, only two studies using the SSS and three studies using the DSAS-1 were included in the current subgroup analysis. Therefore, the results cannot sufficiently reflect the association between diabetes-related stigma and psychological distress under the use of different diabetes-related stigma assessment tools. The moderating effect of the diabetes stigma assessment tool used needs to be tested again in future studies.

The measurement tool used to evaluate diabetes-related distress also significantly moderated the magnitude of the correlation between stigma and distress among patients with diabetes. The correlation coefficient between diabetes stigma and distress was significantly higher when diabetes-related distress was assessed with the PAID scale (r = 0.66) than with the DDS (r = 0.58), PAID-5 scale (r = 0.39) or other instruments (r = 0.36). It is noted that most studies used the PAID scale or DDS to investigate the association between stigma and diabetes distress among people with diabetes. The explanation for this could be due to the excellent psychometric features and widespread use of the two instruments [23, 74]. However, due to some unstable and unbalanced items, the use of the PAID-5 scale may underestimate the correlation between diabetes-related stigma and psychological distress [55]. The significant difference in the correlation between the two variables may be because each scale is separated into distinct dimensions and items, and each scale has a different level of validity and reliability [23, 74]. In addition, because the assessment tools used to evaluate diabetes distress, except the PAID scale, PAID-5 scale, and DDS, were classified as other assessment tools in this study, the relationship between diabetes-related stigma and distress must be further explored to determine whether it is moderated by other less-used assessment tools.

It is worth noting that the measurement tools used to evaluate the levels of diabetes stigma and diabetes distress varied among the included studies. Thus, it was difficult to compare and combine the scores obtained using different assessment tools. In addition, the results of the current study found that the moderating effect of the different measurement tools used on the association of diabetes stigma and diabetes distress was significant. Therefore, further meta-analyses are necessary for adequate studies investigating this subject using the same measurement tool.

Moreover, according to the results of the subgroup analysis, we found no significant influence of the type of diabetes stigma on the overall magnitude of the association. A potential reason might be that a small number of studies (only five studies) exploring the relationship between self-stigma and diabetes-related distress were included in the present meta-analysis, which might influence the result of the moderator analysis. However, the results also identified that self-stigma had a strong correlation with diabetes-related psychological distress; a slightly smaller effect was found for the correlation between perceived/experienced stigma and diabetes-related distress. This finding is consistent with the meta-analytic results provided by Alimoradi et al. [43], who found that the pooled correlation coefficients of the association of self-stigma with psychological distress were slightly larger relative to that of perceived and experienced stigma, which may be a result of acceptance bias and negative stereotypes. Thus, when diabetes stigma is internalized, it may have a stronger effect on diabetes distress. However, due to the small number of studies that explore the association between self-stigma and diabetes distress, the results of moderating role need to be interpreted with caution. Future research should categorize diabetes stigma into different types (i.e., perceived/experienced stigma and self-stigma) and report the relationship between different types of stigmas and diabetes-related psychological distress separately.

According to meta-regression, stigma and psychological distress among people with diabetes mellitus were not significantly moderated by gender. This suggests that the relationship may be stable across genders, although we assumed a stronger association between diabetes-related stigma and distress for females than for males. One potential explanation may be that diabetes-related stigma and distress are very common among people with diabetes [63]. People with diabetes, both men and women, can feel exhausted by the tedium of self-care, treatment regimens, and disease management, distressed by excessive fear of serious complications and shortened life expectancy, and stigmatized by discrimination and prejudice from others [75–77]. Additionally, another meta-analysis similar to this study also found that the association between stigma and mental health was not moderated by gender [41].

Age was shown to have no moderating influence, which was consistent with the result of a previous similar meta-analysis [43]. However, these data contradict the hypothesis, which expected a higher association between diabetes-related stigma and psychological distress in younger people than in older people. One possible reason is that while younger people may be more prone to stigma and prejudice, older people encounter diabetes-related stigma more frequently during their lives. This persistent stigma-related stress and accumulation of negative health outcomes may have resulted in identical consequences for diabetes-related stigma and psychological distress regardless of participant age. In addition, the majority of our included study population was middle-aged and elderly diabetes with a small age span, which may also account for the lack of significant differences in the changes in the correlation between the two variables.

## Limitations and prospects

The current meta-analysis clarified the controversy about the magnitude of the association between diabetes stigma and diabetes distress in empirical studies. However, some limitations still exist in this study. First, because all of the studies included in the analysis used a cross-sectional design, the current findings cannot determine a causal relationship or provide evidence of a temporal relationship between diabetes stigma and psychological distress. Therefore, future related longitudinal studies are necessary to provide more evidence to clarify the temporal association. Furthermore, this meta-analysis was not included in a randomized controlled trial (e.g., whether psychological distress is alleviated after reducing diabetes stigma or vice versa). Hence, the causal relationship between diabetes stigma and psychological distress is unclear. However, there are few randomized controlled trial studies on this topic. Thus, it is encouraged that future randomized controlled studies be conducted to explore the causal association between diabetes stigma and psychological distress.

In addition, because only a few studies were included in some subgroup analyses, the results for the moderating role of some variables should be interpreted with caution. Future research should concentrate on examining moderators to further explore the individual and environmental factors that impact the association of diabetes stigma with diabetes distress. Furthermore, due to the small number of papers, meta-analyses of perceived/experienced stigma and self-stigma in diabetes were not distinguished. Therefore, the results of this study are unable to distinguish the association of perceived/experienced stigma or self-stigma with diabetes distress among people with diabetes. Once there are more adequate studies, further meta-analyses must consider different types of diabetes stigma to explore the magnitude of the association. Finally, statistical methods to evaluate publication bias are only valid when the number of studies is more than ten and the heterogeneity is low. Although we included 19 studies in the present study, the heterogeneity of this meta-analysis seems not to be low. As a result, the present meta-analysis was incapable of detecting all of the publication bias.

## Implications for clinical practice

Clinical health care professionals need to pay full attention to a range of mood changes in diabetes and should be aware of the significant positive relationship between diabetes-related stigma and psychological distress. In addition, health providers can effectively intervene in the stigma of diabetes patients through appropriate strategies to reduce diabetes-specific psychological distress, which contributes to the improvement of quality of life among people with diabetes. For example, the screening of diabetes should be strengthened, effective interventions should be conducted for people at risk of developing diabetes to delay progression to diabetes; the publicity and education of the public about diabetes should be increased, wrong perceptions and prejudice of diabetes should be changed, and psychological counselling and professional psychological construction should be provided for diabetes experiencing stigma. In addition, the medical staff in the process of practical work needs to abandon the intangible prejudice or stereotypes about diabetes mellitus or establish effective communication channels among diabetes patients through group communication and other promotional activities to reduce the risk of diabetes-related stigma and psychological distress.

## Conclusion

In conclusion, diabetes-related stigma and psychological distress are important topics for health care providers to protect the mental health of diabetes. The results of the meta-analysis in the current study suggest that stigma is strongly related to diabetes-related psychological distress. Moreover, the relationship was moderated by diabetes-related stigma and distress measurement tools. Future longitudinal studies should be conducted to reveal the association and causality between stigma and distress in diabetes patients over time. Given the high prevalence and adverse effects of diabetes stigma, health care professionals are encouraged to develop effective and useful strategies to counter or minimize stigma (including self- and perceived stigma) and psychological distress in people with diabetes.

### Electronic supplementary material

Below is the link to the electronic supplementary material.

Supplementary Material 1

Supplementary Material 2

## Data Availability

The datasets used and/or analysed during the current study are available from the corresponding author upon reasonable request.
