# Supplementary material for: The relationship between stigma and psychological distress among people with diabetes: a meta-analysis
Source: BMC Psychol. 2023 Aug 24;11:242. doi: 10.1186/s40359-023-01292-2 (PMC10463375; doi:10.1186/s40359-023-01292-2)
Supplement: Supplementary file 1 — Supplementary Material 1 [file 40359_2023_1292_MOESM1_ESM.docx]

**Supplementary File 1 Searching strategies**

| **Pubmed** | | | | |
| --- | --- | --- | --- | --- |
| #1 | | "Diabetes Mellitus"[Mesh] Sort by: Most Recent | 500,385 | |
| #2 | | (Diabetes) OR (Diabetic) OR (type 2 diabetes) OR (type 2 diabetes mellitus) OR (Diabet*) OR (type 1 diabetes) OR (type 1 diabetes mellitus) OR (Mellitus) | 936,868 | |
| #3 | | #1 OR #2 | 936,868 | |
| #4 | | "Social Stigma"[Mesh] Sort by: Most Recent | 12,500 | |
| #5 | | (Social stigma*) OR (stigma) OR (stigma*) | 53,414 | |
| #6 | | #4 OR #5 | 53,414 | |
| #7 | | "Psychological Distress"[Mesh] Sort by: Most Recent | 6,569 | |
| #8 | | (Emotional Distress) OR (Distress, Psychological) OR (Distress, Emotional) OR (Emotional Stress) OR (Stress, Emotional) OR (Diabetes distress) OR (diabetes-related distress) OR (problem areas in diabetes) OR (diabetes distress scale) OR (distress) OR (diabetes-specific distress) OR (distress syndrome) | 216,683 | |
| #9 | | #7 OR #8 | 216,683 | |
| #10 | | #3 AND #6 AND #9 | 74 | |
| **Web of science** | | | | |
| #1 | | TS=(Diabetes Mellitus OR Diabetes OR Diabetic OR type 2 diabetes OR type 2 diabetes mellitus OR Diabet* OR type 1 diabetes OR type 1 diabetes mellitus OR Mellitus) | 1,511,486 | |
| #2 | | TS=(Social stigma OR social stigma* OR stigma OR stigma*) | 117,331 | |
| #3 | | TS=(Psychological Distress OR Emotional Distress OR Distress, Psychological OR Distress, Emotional OR Emotional Stress OR Stress, Emotional OR Diabetes distress OR diabetes-related distress OR problem areas in diabetes OR diabetes distress scale OR distress OR diabetes-specific distress OR distress syndrome) | 386,362 | |
| #4 | | #1 AND #2 AND #3 | 121 | |
| **Embase** | | | | |
| #1 | | 'diabetes mellitus'/exp OR diabetes OR diabetic OR (type AND 2 AND diabetes) OR (type AND 2 AND diabetes AND mellitus) OR diabet* OR (type AND 1 AND diabetes) OR (type AND 1 AND diabetes AND mellitus) OR mellitus | 1,592,908 | |
| #2 | | 'stigma'/exp OR (social AND stigma*) OR (social AND stigmas) OR stigma* | 74,779 | |
| #3 | | 'Psychological Distress'/exp OR (Emotional AND Distress) OR (Distress AND Psychological) OR (Distress AND Emotional) OR (Emotional AND Stress) OR (Diabetes AND distress) OR (diabetes-related AND distress) OR (problem AND areas AND in AND diabetes) OR (diabetes AND distress AND scale) OR distress OR (diabetes-specific AND distress) OR (distress AND syndrome) | 350,418 | |
| #4 | | #1 AND #2 AND #3 | 164 | |
|  | | **PsycINFO** |  | |
| #1 | | (Diabetes Mellitus) OR (Diabetes) OR (Diabetic) OR (type 2 diabetes) OR (type 2 diabetes mellitus) OR (Diabet*) OR (type 1 diabetes) OR (type 1 diabetes mellitus) OR (Mellitus) | 3,125,049 | |
| #2 | | (Social Stigma) OR (social stigma*) OR (stigma) OR (stigma*) | 257,061 | |
| #3 | | (Psychological Distress) OR (Emotional Distress) OR (Distress, Psychological) OR (Distress, Emotional) OR (Emotional Stress) OR (Stress, Emotional) OR (Diabetes distress) OR (diabetes-related distress) OR (problem areas in diabetes) OR (diabetes distress scale) OR (distress) OR (diabetes-specific distress) OR (distress syndrome) | 899,002 | |
| #4 | | #1 AND #2 AND #3 | 107 | |
| **CNKI** | | | | |
|  | (Subject=Diabetes) AND (Subject=Stigma) AND (Subject=Psychological distress) | | | 3 |
| **VIP** | | | | |
|  | (M=Diabetes OR R=(Diabetes OR Type 1 Diabetes OR Type 2 Diabetes)) AND (M=Stigma OR R=Stigma) AND (M=Psychological distress OR R=Psychological distress) | | | 3 |
| **Wan fang** | | | | |
|  | Subject:(Diabetes) and Subject:(Stigma) and Subject:(Psychological distress) | | | 4 |
