# Supplementary material for: The relationship between stigma and psychological distress among people with diabetes: a meta-analysis
Source: BMC Psychol. 2023 Aug 24;11:242. doi: 10.1186/s40359-023-01292-2 (PMC10463375; doi:10.1186/s40359-023-01292-2)
Supplement: Supplementary file 2 — Supplementary Material 2 [file 40359_2023_1292_MOESM2_ESM.docx]

| **Supplementary File 2**: Results of quality assessment of the cross-sectional studies using an 11-item checklist that was recommended by the Agency for Health care Research and Quality (AHRQ) | | | | | | | | | | | | |
| --- | --- | --- | --- | --- | --- | --- | --- | --- | --- | --- | --- | --- |
| Study | Define the source of information (survey, record review) | List inclusion and exclusion criteria for exposed and unexposed  subjects (cases and controls) or refer to previous publications | Indicate time period used for identifying patients | Indicate whether or not subjects were consecutive if not population-based | Indicate if evaluators of subjective components of study  were masked to other aspects of the status of the participants | Describe any assessments undertaken for quality assurance  purposes (e.g., test/retest of primary outcome measurements) | Explain any patient exclusions from analysis; | Describe how  confounding was assessed and/or controlled | If applicable,  explain how missing data were handled in the analysis | Summarize patient response rates and completeness of data collection | Clarify what follow-up, was expected and percentage of patients for which incomplete data or follow-up was obtained | Total scores |
| Hansen et al., 2017 | Yes | Yes | Yes | Yes | Unclear | Yes | Yes | No | No | Yes | No | 7 |
| Alzubaidi et al., 2022 | Yes | Yes | Yes | Yes | Unclear | Yes | No | Yes | No | No | No | 6 |
| Browne et al., 2017 | Yes | Yes | Yes | Yes | Unclear | Yes | No | No | No | Yes | No | 6 |
| Browne et al., 2016 | Yes | Yes | Yes | Yes | Unclear | Yes | No | Yes | No | No | No | 6 |
| Li et al., 2022 | Yes | Yes | Yes | Yes | Unclear | Yes | Yes | Yes | No | Yes | No | 8 |
| Pedrero et al., 2021 | Yes | Yes | Yes | Yes | Unclear | Yes | No | Yes | No | No | No | 6 |
| Potter et al., 2015 | Yes | Yes | Yes | Yes | Unclear | Yes | Yes | Yes | No | No | No | 7 |
| Hyesun et al., 2022 | Yes | Yes | Yes | Yes | Unclear | Yes | No | No | No | Yes | No | 6 |
| Puhl et al., 2020 | Yes | Yes | Yes | Yes | Unclear | No | Yes | No | No | Yes | No | 6 |
| Holmes-Truscott et al., 2020 | Yes | Yes | Yes | Yes | Unclear | No | Yes | Yes | No | Yes | No | 7 |
| Gredig et al., 2016 | Yes | Yes | Yes | Yes | Unclear | No | Yes | Yes | No | Yes | No | 7 |
| Benioudakis et al., 2022 | Yes | Yes | Yes | Yes | Unclear | Yes | No | No | No | No | No | 5 |
| Polonsky et al., 2021 | Yes | Yes | Yes | Yes | Unclear | No | No | Yes | No | Yes | No | 6 |
| Costabile et al., 2020 | Yes | Yes | Yes | Yes | Unclear | Yes | No | No | No | Yes | No | 6 |
| Wang et al., 2021 | Yes | Yes | Yes | Yes | Unclear | Yes | No | Yes | No | No | No | 6 |
| Joiner et al., 2022 | Yes | Yes | Yes | Yes | Unclear | Yes | No | Yes | No | Yes | No | 7 |
| Kong et al., 2022 | Yes | Yes | Yes | Yes | Unclear | Yes | No | No | No | Yes | No | 6 |
| Li et al., 2021 | Yes | Yes | Yes | Yes | Unclear | Yes | No | Yes | No | Yes | No | 6 |
| Xie et al., 2020 | Yes | Yes | Yes | Yes | Unclear | Yes | No | Yes | No | Yes | No | 6 |
